# Moving From Bilingual Traits to States: Understanding Cognition and Language Processing Through Moment-to-Moment Variation

**DOI:** 10.1162/nol_a_00046

**Published:** 2021-11-11

**Authors:** Lauren K. Salig, Jorge R. Valdés Kroff, L. Robert Slevc, Jared M. Novick

**Affiliations:** Program in Neuroscience and Cognitive Science, University of Maryland, College Park, USA; Department of Spanish and Portuguese Studies, University of Florida, Gainesville, USA; Department of Psychology, University of Maryland, College Park, USA; Department of Hearing and Speech Sciences, University of Maryland, College Park, USA

**Keywords:** bilingualism, executive function, language processing, individual differences, cognitive control

## Abstract

The study of how bilingualism is linked to cognitive processing, including executive functioning, has historically focused on comparing bilinguals to monolinguals across a range of tasks. These group comparisons presume to capture relatively stable cognitive traits and have revealed important insights about the architecture of the language processing system that could not have been gleaned from studying monolinguals alone. However, there are drawbacks to using a group-comparison, or Traits, approach. In this theoretical review, we outline some limitations of treating executive functions as stable traits and of treating bilinguals as a uniform group when compared to monolinguals. To build on what we have learned from group comparisons, we advocate for an emerging complementary approach to the question of cognition and bilingualism. Using an approach that compares bilinguals to *themselves* under different linguistic or cognitive contexts allows researchers to ask questions about how language and cognitive processes interact based on dynamically fluctuating cognitive and neural *states*. A States approach, which has already been used by bilingualism researchers, allows for cause-and-effect hypotheses and shifts our focus from questions of group differences to questions of how varied linguistic environments influence cognitive operations in the moment and how fluctuations in cognitive engagement impact language processing.

## INTRODUCTION

Psycholinguistics research is concerned with developing comprehensive behavioral and neurobiological models of how humans process language at multiple levels of representation. An important aspect of this agenda has been to study how nonlinguistic cognitive systems—such as memory, executive function, and attention—interact with language use. Scientific investigation into such relationships is an attempt to specify how a broader mental architecture may support linguistic procedures, or to reveal what traces variable linguistic experiences may leave on cognitive processing. A conventional approach to this sort of issue is to examine correlated variation in performance in large-scale individual differences studies (e.g., a person with ability X also has ability Y), or to draw comparisons across groups that differ in either ability (as in neuropsychological patients vs. healthy controls) or experience (as in bilinguals vs. monolinguals). The logic behind such methods is that observable associations provide insight into common processes that are shared by the demands of both linguistic and nonlinguistic tasks, which permits inferences about stable relationships among language and cognitive abilities that may be modulated by aggregate experiential contrasts. For example, if bilinguals differ from monolinguals on a particular task of executive function, then the underlying cognitive and neural processes used to complete that task must also be recruited by each population to a different extent while processing language over the course of a lifetime.

In this article, we advocate for a related but distinct approach to these issues, which aims to understand how individuals *compare with themselves*, rather than to other groups, under different information-processing conditions. Akin to traditional group comparisons, the goal is to uncover the functional interplay between language and cognitive systems. But unlike the broadscale group approach, the goal is not to identify cumulative trait differences, but rather to isolate more localized, situational effects such as how linguistic contexts influence cognitive processing and/or (inversely) how manipulations of cognitive engagement influence language processing in the moment. An approach that tests the effects of variable *states* can offer more than correlational data and may allow inferences about when and how language and cognitive processes interact in real time in a causal manner. We believe that such work can provide new insights into the configuration of the bilingual mind and brain that builds on prior research.

We begin by summarizing some findings from past group-comparison research regarding the effects of bilingualism on language and cognition. We then define the Traits and States approaches before turning to some methodological limitations of approaches that examine cognitive abilities as relatively fixed traits to be compared across groups. To address these limitations, we describe a complementary approach in which researchers manipulate either the linguistic context to observe the consequence of these manipulations on cognitive performance (e.g., Adler et al., 2020; Wu & Thierry, 2013) or an individual’s cognitive state to observe its influence on language processing (e.g., Navarro-Torres et al., 2019). By employing a States approach, language scientists can ask questions about how the dynamics of language processing impact other cognitive abilities (and vice versa) on a moment-to-moment basis.

### Brief Summary of Findings From Bilingualism Research on Cognitive Functions and Language Processing

A perennial question in the study of bilingualism is how bilinguals represent their two languages in one mind. Is there a “switch” that completely deactivates one language when it is not currently in use (Costa et al., 1999)? Or are the two linguistic systems interconnected, the effects of which can be observed even in monolingual environments? Findings from bilingual comprehension studies routinely show that words from both languages are simultaneously activated even in single-language contexts, including when a bilingual’s two languages use different scripts or modalities (Hoshino & Kroll, 2008; Marian & Spivey, 2003; Morford et al., 2011; Slevc et al., 2016; Spivey & Marian, 1999; Thierry & Wu, 2007). That is, as spoken words unfold in time, bilinguals do not limit retrieval to the language currently in use, as lexical items from the currently unused language partially compete for selection. These results suggest that there is no clear separation between a bilingual’s two languages; rather, there can be “cross talk” between linguistic representations (e.g., phonological, semantic) for both production and comprehension except in the presence of strong contextual cues (e.g., Chambers & Cooke, 2009; Schwartz & Kroll, 2006).

While bilinguals’ two linguistic systems are not closed off from each other in terms of architecture, they manage to separate their languages in use, rarely failing to use the intended, context-appropriate language. To select the desired word in a particular language, bilinguals presumably must resolve the competition that arises from co-activated representations in both languages. Studies suggest that the unintended language is inhibited when not in use. The more dominant that language is, the more it must be suppressed, resulting in asymmetric switch costs (e.g., Meuter & Allport, 1999, but see Blanco-Elorrieta & Caramazza, 2021, and Declerck & Philipp, 2015, for criticisms of this point). For instance, after using the second language (L2), a bilingual’s first language (L1) is temporarily inhibited and harder to reactivate (e.g., Costa & Santesteban, 2004). This is also illustrated by L2-immersed learners demonstrating increased difficulty producing words in the L1 (Botezatu et al., 2020; Linck et al., 2009). The impact of managing two or more languages may also extend beyond the lexical level, as syntactic parsing strategies in one language can come to impact parsing in another (Dussias & Cramer Scaltz, 2008; Dussias & Sagarra, 2007). For example, Spanish speakers with extensive immersion experience in English as an L2 may begin to parse Spanish sentences with relative-clause attachment ambiguities more like an English speaker; that is, they deploy low-attachment parsing preferences more often than their Spanish monolingual peers or Spanish-immersed bilingual peers. Together, this research demonstrates consistent cross-linguistic influences across a bilingual’s languages over multiple timescales.

In the past three decades, language scientists have asked questions about whether the unique language processing demands associated with bilingualism have cognitive consequences. Namely, they have investigated whether a lifetime of navigating cross-linguistic competition affects aspects of bilinguals’ cognitive processing, including constituents of executive functions: most notably, their ability to resolve competition in nonlinguistic domains. *Executive function* refers to the family of cognitive mechanisms that manage information processing in the brain and flexibly support goal-directed behavior. Some research suggests that bilinguals may have an advantage over monolinguals on conflict detection/resolution tasks (e.g., Flanker, Stroop, and Simon tasks) that test *cognitive control*, a sub-component of executive function that allows individuals to adjudicate among competing representations by promoting task-relevant over -irrelevant information (Abutalebi et al., 2015; Bialystok et al., 2004, 2008; Costa et al., 2009). Neuroimaging findings have implicated neural networks associated with cognitive control—such as the anterior cingulate within medial prefrontal cortex, and subcortical structures such as the caudate and putamen—in bilingual language processing, including relatively greater recruitment in bilinguals as compared to monolinguals (Abutalebi et al., 2015; Luk et al., 2012; Parker Jones et al., 2012; Teubner-Rhodes et al., 2019). Furthermore, bilinguals show less frontal activation than monolinguals for the same performance level on nonverbal cognitive-control tasks, and greater modulation of functional connectivity within frontoparietal networks based on executive-processing demands (see Grundy et al., 2017, for a review). The inference from these findings is that bilinguals are able to deal with *conflict* during information processing more efficiently, perhaps due to greater reliance on other networks, including the language network. Moreover, the finding that bilinguals tend to experience a later onset of dementia symptoms than monolinguals (see Mendez, 2019, for a review) has been ascribed to their neural shift away from relying on frontal regions for executive-function demanding tasks, resulting in greater *cognitive reserve* (Grundy et al., 2017; Mendez, 2019). These results from the Traits approach have improved our understanding of the effects of bilingualism on brain function and its impact on cognitive processing. Overall, however, findings about bilingual advantages in executive functions have been contentious and disparate, with effects emerging in some tasks and contexts but not others (e.g., Bialystok et al., 2009; de Bruin et al., 2015; Hilchey & Klein, 2011; Lehtonen et al., 2018; Paap & Greenberg, 2013; Valian, 2015; Ware et al., 2020).

Mixed findings aside, separating people into monolinguals and bilinguals for group comparisons can address only a subset of questions about the relationship between cognition and language processing. For example, a Traits approach cannot explain *why* and *when* bilinguals may need to engage cognitive control for language processing in the first place, considering the diverse experiences of bilinguals and the range of in-the-moment demands they face. Much of the variation in linguistic experience that makes bilingualism a good test case for questions about language processing is lost when we collapse all bilinguals together into one group. While all bilinguals deal with routine cross-language competition, their processing demands will vary based on their stage of bilingualism (e.g., if they are still acquiring the second language), the specific languages they know, their daily language environment, and the immediate context. For instance, a bilingual who uses one language exclusively at work and the other at home will have different daily processing demands than a bilingual who uses both languages throughout their day, freely code-switching between them in any interactional context (Beatty-Martínez et al., 2020; Green & Abutalebi, 2013). Furthermore, each of these bilinguals will have different processing demands from moment to moment, such as when talking to a monolingual versus to another bilingual who shares their languages. These factors—switching experience, proficiency, exposure, etc.—are exactly the sort of bilingual variation that is proposed to result in different neural and cognitive outcomes (traits) and is likely changed by bilingual experiences in a dynamic rather than a linear manner (DeLuca et al., 2020; Hernandez et al., 2018; Pliatsikas, 2020). While such variation may be considered a nuisance in group comparisons, bilingual variation can guide the questions we ask about language processing in the future if we are willing to take a complementary approach to bilingualism research that overcomes some limitations of the above-described group comparison, or Traits, approach.

## DEFINING THE TRAITS AND STATES APPROACHES

Although the Traits vs. States distinction has only recently begun to be explicitly applied to the development of neurobiological and cognitive models of bilingualism (e.g., Blanco-Elorrieta & Pylkkänen, 2017), the distinction has a longer history in other disciplines. For example, when a clinician takes a measure of an individual’s anxiety, the aim is to measure something about the person and their propensity to be anxious—that is, they hope to measure that person’s anxiety *trait*. However, two people with the same baseline anxiety trait could receive different anxiety scores depending on how their day was going prior to testing or how they react to the assessment situation. For decades, the field of clinical psychology has discussed how to extract stable trait measures from instruments that capture some amalgamation of stable traits, situational impacts, trait-situation interactions, and measurement effects or errors (Kendall, 1978; Steyer et al., 1999, 2015; cf. Allen & Potkay, 1981).

In this article, we consider *states* to be inherently informative, rather than a nuisance variable to be subtracted out of *trait* measures. We will use the working definitions of states and traits provided by the clinical field to distinguish between them in the study of bilingualism. A trait can be considered an “attribute of a person” that guides their behavior while remaining relatively stable across time and situations. On the other hand, a state can be considered an “attribute of a person-in-a-situation” with fluctuations that are “typically reversible and represent systematic but situation-driven variability,” which interacts with baseline traits (Steyer et al., 2015, pp. 72–73).

For our purposes, we consider the Traits approach to be the study of how a lifetime of specific language experience alters an individual’s baseline cognitive abilities or associated, supporting brain structures. When a study measures different conditions of an executive-function task, it usually takes a Traits approach. For example, a study may measure bilinguals’ performance on a Stroop task including congruent trials that require relatively less cognitive control (e.g., saying “red” to the word *red* in red ink) and incongruent trials that demand more cognitive control to resolve conflict (e.g., saying “green” to the word *red* in green ink). If averaged performance on these two different conditions is used to compile an individual’s singular cognitive control “score” or ability, then this is a trait measure. Studies that compare two groups’ scores aim to test for trait differences.

By contrast, we describe the States approach as studying how an individual’s performance fluctuates depending on the given situation. While the range of an individual’s behavioral or neural responses may be limited by their traits, the States approach aims to determine what conditions result in fluctuation within that range. This can be achieved using the same experimental task as studies of traits but designed with modifications to allow for different inferences. For example, a study may ask participants to complete the same Stroop or Flanker task with congruent and incongruent trials in two different linguistic contexts to determine how those contexts affect cognitive-control performance (e.g., higher accuracy and reduced P300 amplitude when ignoring irrelevant words in a mixed- compared to a single-language context; Wu & Thierry, 2013). The opposite direction can also apply: Researchers can use different conditions of the Stroop or Flanker task to manipulate states of cognitive-control engagement in real time and observe their immediate effect on language processing. This type of design alters states to vary the status of moment-to-moment behavioral or neural processes rather than using tasks as psychometric tests that attempt to measure stable traits. Studies that investigate how cognitive or language processing differs within the same individual under different circumstances use a States approach. We propose that modulations in states are as important to understanding the effects of bilingualism on cognitive processing, including executive function, as individual differences in traits.

While a clearly defined difference between Traits and States approaches in bilingualism has not previously been detailed, many others have raised the same limitations of the Traits approach that we will discuss and have called for research practices that we support (e.g., de Bruin, 2019; Grundy, 2020; Luk & Bialystok, 2013; Whitford & Luk, 2019). One goal of this article is to contribute to this ongoing discussion by clarifying the distinction between States and Traits approaches and the questions about bilingualism that each can address. Some have expressed concern about exploring the relationship between bilingualism and executive functions without clear theories (de Bruin et al., 2021). We agree, and thus offer here a theory for how a States approach can result in a clearer understanding of the shared processing demands across linguistic and nonlinguistic behavior.

## LIMITATIONS OF THE TRAITS APPROACH

Research on bilingualism and executive functions has often measured individuals’ performance on a task at one point in time and compared the performance of all bilingual participants to that of all monolingual participants in a sample. This Traits approach led to major advances in our understanding of bilinguals’ efficient reliance on executive functions in bilingual language control, as sketched above. However, a Traits approach necessarily collapses across diversity in language experience within groups and provides only a static illustration of performance in the research lab, thus failing to address the fact that an individual’s cognitive states can also fluctuate and be impacted by context. Given these considerations, we would not want to characterize everyone who has a lapse in performance under laboratory conditions to be “low” in the tested cognitive ability. In this section, we will address these limitations in more detail and discuss how individual differences approaches may not be well-suited for many of the emerging questions surrounding bilingualism and executive function.

### Cognitive and Neural States Fluctuate Within Individuals

#### The traits approach captures only a snapshot of performance

Defining anyone as having *either* good *or* bad executive function is likely a gross oversimplification of how people differ in this way, and adhering to such a strict dichotomy can lead to conceptual and empirical mistakes. A person’s cognitive engagement status naturally fluctuates over the course of a day (Lara et al., 2014) or even throughout a short-term activity such as those used in laboratory tasks. For instance, mind-wandering studies have demonstrated that individuals experience temporary attentional lapses frequently during language comprehension, with some studies reporting that participants had fully on-task attention only about 50–60% of the time (Boudewyn & Carter, 2018a; Smallwood et al., 2008a; Uzzaman & Joordens, 2011). Such attentional fluctuations can influence how much information an individual has collected and retained during their in-the-moment performance (Boudewyn & Carter, 2018a; Smallwood et al., 2008b). One study found that lower levels of alpha oscillatory power at various points during story listening (used as an index of higher attentional engagement) was associated with fewer mind-wandering endorsements at those points and better recall of that information later (Boudewyn & Carter, 2018a). Thus, fluctuations within individuals’ state of *attention control* (another subcomponent of executive function), which regulates the collection of exogenous information from the environment, directly impacts language comprehension.

Various factors can affect whether a person is engaged, including motivation, mood, level of interest in a task, and its degree of difficulty (e.g., Chiew & Braver, 2014). Changes in bilinguals’ linguistic environment may be another such factor. This notion, which we spell out in the Complementary States Approach section, is inspired by mind-wandering studies but also by findings from studies that manipulate linguistic and cognitive states in monolinguals. Such studies are motivated, in part, by theoretical accounts that postulate that during language comprehension, readers and listeners often encounter conflicting cues to interpretation that can generate misanalyses and the need to revise, which relies on cognitive control to prevent comprehension from running aground (Novick et al., 2005). For example, moment-to-moment variation in the transient state of cognitive control, which detects and resolves conflict during information processing, impacts the ease with which readers and listeners revise misinterpretations of linguistic input in real time (Hsu & Novick, 2016; Hsu et al., 2021; Thothathiri et al., 2018). Likewise, how easy or difficult a sentence is to understand—arguably a modulation in linguistic state—recruits varying levels of cognitive-control procedures dynamically (Kan et al., 2013). These findings have been interpreted within classic models of cognitive control, in which the detection of conflict guides processing toward the most informative cues in the input to maximize performance and decrease errors (Botvinick et al., 2001). Botvinick and colleagues’ classic conflict monitoring account of cognitive control (2001) describes a model exclusively in terms of states, not individual traits—focusing on *how* and *when* cognitive-control mechanisms engage to bias information processing toward currently-relevant over -irrelevant cues in the service of optimizing performance. Such models can explain the findings briefly summarized here. We advocate for a return to this sort of theorizing, concentrating for example on how aspects of bilingual language processing vary the engagement status of different cognitive systems (and why).

Studies that focus exclusively on whether a person with strong cognitive control (i.e., the ability to promote task-relevant cues in the service of conflict resolution) performs differently than a person with weak cognitive control on some other task—a trait contrast—capture only a snapshot of individuals’ performance in time. Trait-based approaches miss important data about how natural oscillations in attention or cognitive control impact behavior. Consider, for instance, traditional comparisons between neuropsychological patients with cognitive-control deficits and college students, who look starkly different in how they interpret language. Healthy young adults are better than people with brain damage at revising misinterpretations of language input, which involves cognitive control (Novick et al., 2005, 2009; Vuong & Martin, 2011). Yet, other studies show that manipulations of healthy young adults’ cognitive control—increasing it or decreasing it, either through neurostimulation or behavioral means—immediately affects their language processing (Hsu & Novick, 2016; Hsu et al., 2021; Hussey et al., 2015). A healthy person’s sentence interpretation can come closer to resembling that of a patient with a cognitive-control disorder by temporarily downregulating their relative cognitive-control engagement. The standard categories therefore do not uniformly apply in any obviously fixed manner: A college-student participant does not fit tidily into a certain bin of cognitive-control ability because that ability gets mobilized to a different extent depending on its state of engagement, with noticeable consequences on linguistic behavior. These state-based data permit the inference that modulations in cognitive control *cause* modulations in language processing. There are two noteworthy points: (1) Such shifting performance patterns will be missed when classifying people categorically by their cognitive control “score,” and (2) causal inferences cannot be drawn from individual-to-individual or group-by-group correlations, in which such relationships could be mediated by additional, unmeasured factors.

#### Linking hypotheses and the logic of the traits approach

Additionally, studies that assess cognitive abilities as traits may be pushing the limits of what can be reasonably concluded from the tasks used. One issue in this realm is the varied definitions applied by different researchers to “executive functions.” Hypotheses about how executive functions play a role in bilingual language processing should clearly articulate which *components* of executive function are being measured, with a careful delineation of how such components are shared across linguistic and nonlinguistic task demands. In other words, it is insufficient to suggest that “attention control” or “inhibition,” for instance, are required for tasks X and Y without stepping through a chain of inference—a linking hypothesis—that connects how the necessary procedures for tasks X and Y are recruited as the relevant system processes a stimulus in real time. Without these specifications, attempts to correlate abilities across different tasks run the risk of Type II errors: for example, concluding that a certain cognitive process is not involved in some linguistic process (when in fact it is) because the groups do not differ, or clear patterns of individual differences do not emerge.

Consider the case of conflict trials from the color-word Stroop task (e.g., saying “blue” to the word *red* written in blue font) and the arrow Flanker task (e.g., → → ← → →), both of which are often used as measures of inhibition in some executive-function frameworks. Although they contribute to a common factor in latent variable analyses, they often fail to correlate (Friedman & Miyake, 2004; Gärtner & Strobel, 2021; Hedge et al., 2018; Unsworth, 2010), which could generate possibly unwarranted conclusions about domain-specificity, e.g., inferring that there are separate inhibition mechanisms for verbal and nonverbal tasks. Problems with interpreting null results aside, there are theoretical issues. For performance to correlate across tasks, one must assume that the same *degree* of some executive-function process (here, inhibition) is similarly required across the two tasks, which is a dubious premise. For instance, the representations one must overcome during Stroop, on account of a lifetime of reading, are arguably stronger than those in a relatively novel Flanker task. This means that even someone with a stable inhibition ability will have to apply inhibition to different extents across those two tasks (more for Stroop, less for Flanker). For the inhibition signal to correlate in magnitude, we must estimate approximately equal representational strengths, which, in this example, is likely false (for more discussion, see Nozari & Novick, 2017, and Hsu et al., 2017). Thus, correlational failures might reflect actually different mechanisms required for the two tasks, an ill-defined linking hypothesis, and/or a shared process that goes undetected because it need not be implemented to the same extent and so correlated variation is not observed (and should not be expected).

As we will discuss further below, there are also theoretical reasons to expect that certain executive functions will be upregulated more by particular bilingual experiences than others (Green & Abutalebi, 2013). Even factoring out the nuances of bilingualism, executive functions are not a monolith (Miyake et al., 2000), and spelling out the distinct procedures that are engaged across different tasks may offer novel insight into the processing demands of certain linguistic tasks. For example, in studies of monolingual language processing, manipulations of cognitive control shape the ease with which listeners resolve temporarily ambiguous sentences and recover from misinterpretation, whereas manipulations of attention control do not (Hsu et al., 2021). Attention control and cognitive control are terms that are frequently used interchangeably in psycholinguistics research, but in fact they may reflect related but independent constituents of executive function. Whereas *attention control* refers to the ability to orient to, and collect information from, sensory input in the exogenous environment (Petersen & Posner, 2012; Posner & Petersen, 1990), *cognitive control* refers to the ability to characterize (and recharacterize) the input once it has been collected at some endogenous level of representation (e.g., Botvinick et al., 2001). Indeed, attention control can remain engaged over sustained periods of time, whereas cognitive control appears to operate more transiently, as it is needed. Moreover, there are different neural markers of attention and cognitive control (e.g., oscillatory power in the alpha and theta bands respectively; see Boudewyn & Carter, 2018b, and Rommers et al., 2017). Failure to consider such definitional, empirical, and neurobiological contrasts, alongside failure to articulate clear and consistent linking assumptions, may yield misunderstandings about when and how performance across tasks should be expected to interact, and also about the independent roles that different sorts of executive functions might play during language processing.

Moreover, there are important statistical considerations: Tasks such as Flanker and Stroop that are intended to measure executive functions such as response inhibition are not always strongly correlated with each other, perhaps indicating that they may be tapping into distinct abilities (Hedge et al., 2018), although latent variable analysis indicates they are related (Unsworth, 2010). As argued above, performance may fail to correlate because task-specific representations may not require inhibition to the same extent (Nozari & Novick, 2017).

Some of these commonly used tasks, furthermore, have been shown to have low test–retest reliability, in part because of minimal between-participant variance. That is, while these tasks can be good for manipulating people’s relative state of cognitive engagement (e.g., through congruent vs. incongruent trials), they may not be stable enough to measure individual differences—and in fact, the robust effects these tasks elicit are part of what makes them ill-suited to looking at individual differences (Hedge et al., 2018). This statistical issue can limit group comparison approaches to executive functions even when taking an individual differences approach but highlights the strength in using executive-function tasks to measure in-the-moment variation within (rather than among) individuals (Whitford & Luk, 2019).

#### Language affects cognitive/neural states

The findings discussed here indicate that relying upon the snapshot approach alone tells only part of the story. Further, they suggest that it is important to consider the factors that affect relative states of cognitive engagement in the moment, and how such dynamic fluctuations shape behavior in that same moment. Recent research in bilingualism has adopted such an approach by examining fluctuations of cognitive engagement under different linguistic contexts. Under this States approach, tasks like Flanker are not used as a stable individual difference measure but instead as a manipulation or measurement of cognitive control on a moment-by-moment or blocked (contextual) basis, thus circumventing some of the limitations listed above and leading to interesting findings. For example, emerging data indicate that bilinguals’ cognitive-control engagement, as measured by Flanker performance, is adjusted in real time depending on the linguistic context. Contexts that involve language mixing (i.e., the presence of both languages within an experimental session) or code-switching modulate bilinguals’ Flanker performance compared to single-language input, allowing inferences that such contexts affect the engagement status of cognitive control (Adler et al., 2020; Wu & Thierry, 2013). The notion of *language modes*, popularized by Grosjean (1985, 1998, 2001), means that people shift into different states of language expectancies based on their assumptions in any given context. These contexts create different processing demands, which affect behavior in various ways.

Recent work demonstrates that this shift between language modes can dynamically affect neural activity in the dorsolateral prefrontal cortex (dlPFC) and anterior cingulate cortex (ACC), areas implicated in bilingual language control and cognitive control more generally (e.g., Braver et al., 2003; Crinion et al., 2006). Blanco-Elorrieta and Pylkkänen (2017) examined switch costs as measured by reaction time and MEG in production and comprehension within the same group of Arab-English bilingual participants. The tasks varied from artificial externally cued language switching paradigms commonly used to examine bilingual language control to more ecologically supported naturalistic switches, while also varying whether imagined interlocutors were monolingual or bilingual. The greatest switch costs and neural activity in dlPFC and ACC were observed in the artificial, pragmatically unsupported task and diminished in the more naturalistic tasks. Considering the different processing demands elicited by a particular language mode offers information that is distinct from snapshot tests of cognitive traits, in which we are capturing and averaging a person’s behavior throughout a context or experimental setting, often without acknowledging what language mode they may be in.

### Bilinguals and Monolinguals Are Not Monoliths

#### Bilinguals experience different interactional contexts

Given that bilinguals may engage different linguistic and cognitive processes dependent on context, it is also important to consider what language mode(s) bilinguals experience on a regular basis. Bilingual experiences differ in myriad ways. For example, some bilinguals are likely to maintain a clear, functional separation between languages throughout their day (Language A for work; Language B for home); others may constantly switch between their languages based on the relationships that they maintain with other people (Language A with Person A; Language B with Person B; Languages A, B, and spontaneous code-switching with Person C); while still others may switch freely between their languages with all members of their community (Languages A and B and spontaneous code-switching with everyone; Green & Abutalebi, 2013). Even if a bilingual spends most of their time in one of these modes, their language mode or cognitive state can still vary based on context, making it important to consider both differences between bilinguals and the current state a bilingual is in.

Each distinct interactional context may impose different processing demands that require bilinguals to recruit different cognitive processes, and it is crucial that we consider these varying demands both among and *within* bilinguals. Green and Abutalebi’s Adaptive Control Hypothesis (2013) proposes that varying interactional contexts allow bilinguals to develop different strategies in regard to their executive-control processes. For example, a bilingual who uses two languages in one context, but each with a different person, may develop better moment-to-moment task (dis)engagement skills, while a bilingual who uses a single language in each interactional context might develop goal-maintenance skills, and a bilingual who freely code-switches with everyone might benefit from opportunistic planning abilities. Indeed, one study found that Spanish-English bilinguals from environments with different default interactional contexts (a separated language context in Spain, an integrated language context in Puerto Rico, and a varied context in the United States) differed in their performance on the AX-continuous performance task (AX-CPT), which measures proactive and reactive control processes (Beatty-Martínez et al., 2020). As the authors of that article point out, “by most past accounts, the bilinguals from the three contexts examined here would be assumed to represent samples drawn from the same or similar underlying populations of proficient Spanish–English speakers” (p. 20). Alongside variable patterns across bilinguals’ proficiency levels, ages of acquisition, etc., these observations suggest that it is inappropriate to treat “bilingual” as a single, indivisible category, even when considering the specific language pair of bilingual participants.

#### Specific bilingual experience affects processing

Theories of bilingual language processing have also moved in the direction of considering the various demands of bilingualism. While the Adaptive Control Hypothesis (Green & Abutalebi, 2013) carefully considers how different bilingual language experiences modulate processing and cognitive-control demands, the hypothesis reflects cumulative experience under distinct interactional contexts, thus representing a Traits approach. Green and Wei (2014) instead focus on the language control demands of different types of bilingual speech (Muysken, 2000). This Control Process Model of Code-switching proposes distinct states of speech planning (competitive or cooperative) based on the contextual demands and intentions of bilingual speakers. Single language speech planning requires constant suppression of a non-target language, thus invoking a competitive state. In contrast, code-switching falls under cooperative planning even though different types of code-switches (e.g., single word or copious switches across languages) invoke different control states (coupled vs. open control, Green & Wei, 2014). Both models suggest that a bilingual’s dominant mode for interactional contexts will affect their cognitive strategies in a fixed way, although acknowledging that each bilingual may shift their mode of control as needed in particular situations. Indeed, both of these hypotheses attribute fixed cognitive differences for different types of bilinguals to their (at least initial) cognitive flexibility to adjust to the language demands of their dominant interactional context. This flexibility is demonstrated by the adjustments that bilinguals and/or L2 learners’ linguistic systems make in immersion contexts (Botezatu et al., 2020; Dussias & Sagarra, 2007; Linck et al., 2009). Recently, Green (2018) has expanded the Control Process Model to account for more moment-to-moment changes in language control demands required for bilingual code-switching, shifting to a more states-based hypothesis. As we will demonstrate in the next section, language scientists have been pulling on ideas from these hypotheses and increasingly applying their logic in States approaches that consider both an individual’s prior experiences and in-the-moment demands.

Accounting for different bilingual experiences—from interactional context to age of acquisition—is also important when considering the potential neurological impacts of bilingualism. DeLuca and colleagues recently proposed the UBET model, which outlines how varied language experiences may affect distinct control processes and, in turn, supporting brain structures (DeLuca et al., 2020). For example, the UBET model proposes that a bilingual’s diversity of language use and language switching experience place variable demands on executive-control processes, resulting in cognitive changes to proactive control and neural changes to cortical grey matter and frontal activation. Factors such as proficiency and duration of use are posited to have distinct cognitive and neural effects. Just as the UBET model supports different outcomes for different bilingual experiences, Pliatsikas’s Dynamic Restructuring Model (2020) supports different restructuring of gray and white matter based on the quantity and quality of language experience. Importantly, these changes are not linear or incremental; rather, initial increases in cortical and subcortical gray matter volume at an early stage of bilingualism may be reduced at later stages of bilingualism, perhaps as connections are pruned to be more efficient. Hernandez and colleagues’ framework of Neuroemergentism (2018) further supports the idea that bilingualism has nonlinear, dynamic effects on cognition and neurology. For example, age of acquisition may affect language representations in the basal ganglia, and the resulting parasitic or symbiotic relationship between the L1 and L2 will dynamically affect use of the prefrontal cortex. All of these models reflect the increasingly apparent conclusion that bilingualism is not a singular category with uniform impacts on architectures and mechanisms for information processing. Clearly defining and quantifying bilingualism or the language variable of interest can help clarify what effects we expect to see in particular individuals under different circumstances.

#### Diversity of experience beyond bilingualism

Although we have focused on the diversity of bilinguals, by pitting groups against each other, traditional group-comparison approaches in bilingualism research may be masking variation within even the monolingual group. Monolingual experiences also differ, yet group-level comparisons may focus on a particular version of monolingualism in participant samples. While studies may include participants who are functionally monolingual (i.e., they would classify themselves as monolingual), their experiences with a single language may vary. For instance, bidialectal monolinguals whose two dialects are quite different in terms of lexicon as well as grammatical and phonological rules (e.g., African American English vs. mainstream American English) may engage in similar code-switching behaviors as bilinguals—shifting their language use between speakers and contexts as necessary. This type of monolingual experience likely also results in different cognitive demands and routines (e.g., Kirk et al., 2018; Oschwald et al., 2018; cf. Poarch et al., 2019; Ross & Melinger, 2017).

Additionally, it is certainly possible that nonlinguistic factors such as particular types of training or expertise might contribute to individuals’ performance on language processing or executive-function tasks. As one example, musical training may affect individuals’ performance on specific executive-function tasks (Moreno et al., 2011; Okada & Slevc, 2018). And cognitive-control practice over the course of weeks can affect performance not only on the trained nonlinguistic task (e.g., N-back with lures), but also on language-processing measures that have overlapping conflict-processing demands on the trained task (e.g., Hussey et al., 2017; Novick et al., 2014). Presumably, these lab-based effects are miniature versions of the kinds of effects that emerge through lifelong experience. Just as bilingualism can affect the brain and behavior through experience-based plasticity (e.g., Pliatsikas et al., 2020), so can other learning experiences. We do not always know each participant’s history with these types of experiences, and the cumulative impact of lived experiences could give the group comparison “edge” to either the monolingual or bilingual group. Given the difficulty of controlling for each possible source of participant variation, it is beneficial to compare participants to themselves under different conditions rather than making group generalizations.

#### Statistical issues and quantifying bilingualism

Clearly, there are various types of bilinguals and monolinguals. While our field has made great strides from separating these diverse populations into two (simplified) groups, treating continuous variables as dichotomous ones is a practice fraught with empirical and theoretical problems (Whitford & Luk, 2019). Dichotomizing variables that are actually continuous can mask individual differences, reduce effect size and power, and hide nonlinear relationships, among other statistical issues (see, e.g., MacCallum et al., 2002). Given the impact on individual difference detection, it is particularly important that we do not unnecessarily dichotomize variables when we are using language processing methods that already struggle to detect individual differences in our effects of interest (e.g., the garden-path ambiguity effect in self-paced reading; Cunnings & Fujita, 2021; James et al., 2018).

Thus far, it is clear that monolinguals and bilinguals can vary in their language experience in ways that are important to capture on a continuum; however, it is not always clear how to quantify that difference from the language history measures we collect. Recent work provides one option, called *language entropy*, whereby researchers can estimate a bilingual’s diversity of language use both as a function of their day-to-day language routines and across various social contexts (Gullifer & Titone, 2019). For example, higher language entropy, or greater diversity in language experience, relates to greater resting-state brain connectivity between regions associated with second language speech production and executive functions (e.g., between the bilateral putamen and ACC; Gullifer et al., 2018). Another fruitful avenue is to incorporate methods from social-network analysis to further quantify the nuances of bilingual language experience (Tiv et al., 2020) and demonstrate its effect on language processing (e.g., Lev-Ari, 2018, Lev-Ari & Shao, 2017). Using an exposure-based approach to quantifying bilingualism, one study drew different conclusions and interpretations about the effects of bilingualism and age on executive function than studies that treat age and bilingualism as dichotomous (Incera & McLennan, 2018). Unfortunately, many studies on bilingualism do not report objective measures of participants’ proficiency or language usage (Surrain & Luk, 2017).

Having to quantify bilingualism can help us specify exactly what aspect of bilingualism we expect to have an effect on a given measure: Is it proficiency? Age of acquisition? Amount of exposure to each language? Code-switching frequency? In doing so, we may find that people previously labeled as monolingual actually demonstrate aspects of bilingual language processing to some degree. This could be due to mislabeling of a sample. As Grundy points out in a recent analysis (2020), there is no standardized method for splitting the continuum of bilingualism into “monolinguals” and “bilinguals,” and participants labeled as part of the “monolingual” group sometimes have experience learning an L2. Conversely, even “true” monolinguals may look more like bilinguals after short-term learning experiences. For instance, one study found that even beginner language learners showed a (small) cognate facilitation effect in electrophysiological measures (Bice & Kroll, 2015). Another study found N400 differences in event-related potential (ERP) responses to words versus nonwords in L2 learners after only 14 hours of instruction (McLaughlin et al., 2004). Differences in linguistic diversity in the environment may also impact how monolinguals process or learn a new language: Monolinguals surrounded by a greater diversity of other languages (irrespective of their knowledge of those languages) showed marginally significant late anterior positivity ERP to novel words versus nonwords in a newly learned language that monolinguals from a more homogenous community did not demonstrate (Bice & Kroll, 2019). Within monolinguals’ native language, increased exposure to infrequent structures influences the ease with which monolinguals process difficult sentences, such as object relative clauses (MacDonald & Christiansen, 2002; Wells et al., 2009). And persistent experience with the grammatical environments in which particular verbs appear continuously shapes the verb biases that individuals form to guide comprehension in real time (Ryskin et al., 2017). Thus, it can be important to understand the background of participants when drawing comparisons among them.

#### Bilingualism remains informative when treated as a continuum

In placing language experience on a continuum—where resolving competition and misanalysis through domain-general control processes (even in monolinguals) is the norm—we might ask: What is special about bilingualism? Why might certain aspects of bilingualism lead to qualitative and/or quantitative differences with monolingual language experience? Here, we may again turn to different experiences in bilingualism for insight. The majority of bilinguals are unimodal bilinguals, meaning that they negotiate competition within and across languages through a single modality. In contrast, bimodal bilinguals, such as speech-sign bilinguals, are not constrained to a single modality (Emmorey et al., 2008a). If competition for selection within the same modality is one underlying cause of increased reliance on and efficiency in cognitive control, then bimodal bilingualism may place different demands on bilingual language use (Blanco-Elorrieta et al., 2018; Emmorey et al., 2008b), despite the continuing presence of bilingual language co-activation across modalities (e.g., Morford et al., 2011; Pyers & Emmorey, 2008). Along similar lines, unimodal bilinguals are constrained to producing code-switches, whereas bimodal bilinguals can additionally engage in code-blending, in which both languages are produced at the same time across modalities (e.g., spoken and signed). We can gain insights into the processing demands of bilingual language practices by examining bimodal bilinguals who have been observed to engage in code-blending over code-switching (Emmorey et al., 2008a), thus suggesting a biological constraint to unimodal code-switching despite apparent costs.

Similarly, we can capitalize on different modalities of bilingualism to learn about bilingual language control in general. For example, an MEG study investigated bimodal bilinguals in two different states: transitioning from code-blending to using one language (“turning off” a language) and transitioning from using one language to code-blending (“turning on” a language), aspects of bilingual language control that simply cannot be dissociated in unimodal bilinguals. Increased activity in bimodal bilinguals’ ACC when turning off but not turning on a language suggests disengaging the no-longer-desired language is the more difficult aspect of bilingual language control for bilinguals in general (Blanco-Elorrieta et al., 2018). In turn, these insights from bimodal bilingualism suggest that bilingual code-switching is actually a skillful language practice since bimodal bilinguals show a distinct preference for code-blending over code-switching. Yet not all bilinguals engage in frequent code-switching and consequently demonstrate differences in how they process code-switches in comprehension (Beatty-Martínez & Dussias, 2017; Valdés Kroff et al., 2018). Interestingly, the P300, an early frontal positivity component in EEG, is found to be modulated based on the self-reported code-switching behavior of bilinguals and may be reflective of a switch cost for nonhabitual code-switchers needing to shift from a “narrow” attentional state that supports unilingual processing to a “broader” attentional state to accommodate code-switching (Beatty-Martínez & Dussias, 2017; Valdés Kroff et al., 2020). In sum, treating bilingualism as a continuum does not make studying its effects less interesting; it simply opens us up to studying a wider range of bilingual factors and their effects (be they qualitatively or quantitatively different than effects of monolingual experience) on various cognitive abilities and neural structures.

Given the methodological, statistical, logical, and theoretical concerns involved when comparing bilinguals with monolinguals, we suggest that researchers should engage in a vigorous inquiry into how bilinguals compare *with themselves* under different language-processing conditions and observe the real-time consequences of these subtle manipulations. As we will detail below, such an approach circumvents many of the concerns outlined here (see Box 1 for example limitations of the Traits approach).

**Box 1.**  Sample limitations of the Traits approach

**Table:** 

Limitations related to executive functions	Does not determine a person’s level of engagement at the time of measuring their executive function.
	Does not allow for cause-and-effect claims about how language processing affects executive-function recruitment (or vice versa).
	Uses executive-function tasks that can have low test–retest reliability.
	Uses group comparisons, which makes it difficult to control for all possible experiential, cultural, and demographic factors that affect executive functions.
Limitations related to bilingualism	Groups bilinguals from different interactional contexts together even when they may recruit executive functions differently.
	Cannot capture moment-to-moment shifts in bilinguals’ control processes based on language context.
	Does not consider diverse monolingual experiences that could differentially impact executive functions (e.g., bidialectalism).
	Treats the continuum of bilingualism as a dichotomy, which has statistical and theoretical drawbacks.
	May incorrectly classify people with minimal (but critical) second-language experience as monolinguals.

## A COMPLEMENTARY STATES APPROACH

In view of the limitations sketched above, it is incumbent upon language scientists to ask new questions that are not about trait advantages that compare bilinguals to monolinguals. When bilinguals have faster response times, higher accuracy, or more efficient behavioral or neural activity, it may be due to bilinguals’ specific language backgrounds and/or to the immediate testing context. Performance on a task at one point in time may not always reflect a lasting advantage so much as an ability that has been temporarily upregulated at the time of testing due to processing demands, linguistic or otherwise. In cases where routine linguistic processing demands *have* resulted in different cognitive strategies at some baseline, we should be careful not to think of this as a bilingual-wide advantage, but rather as a cognitive adaptation to particular language demands that may also be present for certain monolinguals (e.g., monolinguals who are bidialectal, who navigate relatively more linguistic conflict, or who are exposed to more linguistic diversity).

Instead, we can turn to questions such as: What properties of bilingualism might engage cognitive control or other executive functions in the first place? Are there unique language-processing demands that bilinguals face in real time that might regulate attention and cognitive control? Rather than comparing bilinguals to monolinguals, these questions focus on the specific demands of bilingualism and how linguistic and cognitive systems meet those demands. The answers to these questions could eventually have significant implications for pedagogical approaches and educational decisions by revealing how subtle manipulations of bilingual behavior (e.g., interpreting a code-switch vs. not) recruit attention and cognitive-control mechanisms (Antón et al., 2016; Mallikarjun et al., 2017). Such an approach therefore assumes a more dynamic process at play rather than cumulative differences. Bilinguals’ experiences vary widely, and their respective environments may place different pressures on cognitive systems over time that may not apply uniformly across all bilinguals as a fixed trait and, indeed, may not always result in default-state benefits to those systems. Consistent with these ideas, a recent Bayesian meta-analysis found evidence to support executive-function advantages in bilinguals over monolinguals but noted that such differences are on the order of milliseconds and may depend on factors such as bilinguals’ interactional context (Grundy, 2020). Just as Grundy suggests in that article, we are advocating for more research on *when* (not if) bilinguals’ language processing recruits executive functions, with well-articulated linking assumptions.

Grundy’s analysis included 167 studies from the 2000s on bilingualism and executive functions, all of which took a group comparison (i.e., Traits) approach (Grundy, 2020). Relatively speaking, there are far fewer bilingualism studies taking a within-participant States approach, although some recent work has moved in that direction. Having learned a lot—but also left with lingering questions—from past work using the traditional Traits approach, researchers have increasingly been considering the diversity of within-language experiences and contexts. For instance, recent studies have examined how same language-pair bilinguals from different communities use language differently and/or perform differently on cognitive ability tasks (e.g., Beatty-Martínez et al., 2020; Blokzijl et al., 2017; Hofweber et al., 2016, 2020). Other work has tested how bilinguals’ experiences with particular production distributions correlate with their ease of comprehension (Beatty-Martínez & Dussias, 2019; Rossi et al., 2021; Valdés Kroff et al., 2017, 2018) or their performance under different task demands (Blanco-Elorrieta & Pylkkänen, 2017; Gollan & Ferreira, 2009). These types of approaches are a great starting point for understanding the effects of different bilingual language experiences.

### Past Work Applying a States Approach to Bilingualism and Cognition

#### Manipulating states within experimental blocks

For the purposes of testing for relationships between language processing and cognition, we have argued that within-bilingual comparisons allow us to answer questions about the moment-to-moment demands of these systems and how they interact. Take, for example, a study in which bilingual participants completed a Flanker task testing their cognitive control while ignoring words interleaved between the trials (Wu & Thierry, 2013). When words from both their languages were mixed with the Flanker task, bilinguals made fewer errors and had a reduced P300 ERP mean amplitude on incongruent, conflict-laden Flanker items as compared to a block when words from only one of their languages were interleaved. By observing the same bilinguals in different linguistic contexts, they were able to conclude that a mixed-language situation upregulated cognitive control more than a single-language situation, demonstrated by performance modulations on the nonlinguistic Flanker task.

Similarly, Jiao and colleagues found that Chinese-English bilinguals performed better on incongruent and congruent Flanker trials when they were interleaved with reading or listening comprehension tasks that appeared in both languages as opposed to in a single-language block (Jiao et al., 2019). In follow-up experiments using a non-conflict version of the Flanker task, the effect disappeared, suggesting that the mixed-language context upregulated conflict monitoring rather than general alertness. In another study, incongruent and congruent Flanker trials were interleaved with a picture-naming task that was completed in either one language or both languages throughout a block. Although a block’s language context did not affect behavioral performance on the Flanker task, using ERPs the authors found that a mixed-language context resulted in larger N200, smaller P300, and smaller late positive component (LPC) mean amplitudes on Flanker trials when compared to single-language contexts (Jiao et al., 2020). Using a States approach that compared bilinguals to themselves under different linguistic conditions, the authors concluded that the mixed-language context resulted in better efficiency on the task, perhaps due to upregulated conflict monitoring. Using a similar blocked language context approach with fMRI, Guo and colleagues (2011) manipulated the language demands of a picture naming task to draw conclusions about the brain regions involved in distinct inhibitory processes. They determined that bilinguals engaged the ACC and supplementary motor area relatively more in the mixed-language block versus single-language blocks, which they took as a measure of local inhibition of specific other-language representations. In general, these studies show that different language contexts engage distinct executive-function processes within bilinguals.

While the aforementioned studies manipulated language context across blocked sessions to observe its effect on a measure of executive function, it is also possible to design the opposite paradigm by manipulating the type of conflict across blocked sessions to observe the effect on language processing. In one such study, Chinese-English bilinguals completed a Stroop ink color naming task where the words were color words in either language (conflict block) or non-color words in either language (no-conflict block). Bilinguals took longer to name the ink color when switching between languages from trial to trial (as compared to staying in the same language) in the conflict block as compared to the no-conflict block, especially when switching into the L1 (Liu et al., 2019). Comparing bilinguals with themselves in this way revealed that general conflict-resolution or inhibition demands can affect bilinguals’ language processing in the moment.

#### Manipulating states from trial-to-trial

A finer-grained approach is to investigate such effects at the trial level rather than in a more global block context. This can be done by manipulating linguistic demands (e.g., interpreting a code-switched sentence vs. a unilingual sentence) to observe the effects of such manipulations on cognitive performance on a trial-by-trial basis. One such method leverages a recognized effect in cognitive psychology known as *conflict adaptation*, where conflict trials (e.g., → → ← → → ; incongruent trials of a Flanker task), relative to no-conflict trials (e.g., → → → → → ; congruent trials of a Flanker task), strengthen performance on subsequent conflict trials (e.g., Gratton et al., 1992; Ullsperger et al., 2005). This indicates that conflict detection engages cognitive control, which sustains to facilitate processing of ensuing conflict on subsequent trials by meeting task demands. Taking a cross-task conflict adaptation approach, one study found that bilinguals responded faster to incongruent Flanker trials after reading a code-switched sentence as compared to a unilingual sentence (Adler et al., 2020). In a similar design, Bosma and Pablos (2020) found that the difference in P300 amplitude between congruent and incongruent Flanker trials was modulated by a code-switch in a prior trial. These studies’ findings are in line with others’ conclusion that dual-language contexts engage cognitive control (Jiao et al., 2019; Wu & Thierry, 2013). Despite ostensible differences in stimulus features between Flanker and sentence trials, the manipulation of switching condition (code-switched vs. unilingual sentences) shifted performance on Flanker trials with conflicting cues. One interpretation of this result is that the shared need for conflict resolution in both tasks allows adaptation between them: Integrating a code-switch during comprehension engages a common cognitive-control process, which remains engaged to accomplish optimized performance on incongruent Flanker items. Specifically, this suggests that integrating a code-switch during real-time comprehension deploys domain-general cognitive-control procedures to a different extent than unilingual sentence processing, presumably to resolve the conflict that arises across linguistic representations. Thus, interpreting a code-switch represents a different linguistic state than interpreting an otherwise matched unilingual sentence, resulting in transient changes in cognitive-control recruitment.

The conflict-adaptation approach has also been implemented to observe whether the relative engagement status of a bilingual’s cognitive-control state influences their real-time language processing, in particular, their ability to recover from temporary misinterpretations (Navarro-Torres et al., 2019). To manipulate cognitive control, one study interleaved congruent and incongruent Stroop trials with a language task in which participants had to follow instructions that were either unambiguous or temporarily ambiguous and ripe for misanalysis (e.g., “Put the frog on the napkin onto the box,” where “on the napkin” is initially interpreted as a destination but must be revised once “onto the box” unfolds, conflicting with that interpretation; Navarro-Torres et al., 2019). When cognitive control was upregulated after an incongruent Stroop trial, eye-fixation data showed that bilinguals revised earlier as compared to when cognitive control was comparatively less engaged (i.e., the prior trial was a congruent Stroop trial). This replicates findings in monolinguals (e.g., Hsu & Novick, 2016; Hsu et al., 2021; Thothathiri et al., 2018) and demonstrates how parsing and interpretation can be rapidly affected by different states of cognitive engagement. Moreover, when comparing the effect of variable states in bilinguals to monolinguals, the data revealed that bilingual listeners began disambiguating the ambiguous sentences earlier than monolingual listeners, suggesting group differences in the timing of cognitive-control engagement (Navarro-Torres et al., 2019). This study exemplifies the value that a combined States and Traits approach can have, especially for considering the effects of both immediate state-based effects and lifetime experience.

Together, these studies demonstrate that bilinguals’ cognitive-control procedures are not static and that different language processing demands mobilize them to different extents. The findings also begin to converge on hypotheses about *how* certain bilingual demands (e.g., processing a code-switch during comprehension, a situation in which a bilingual’s two linguistic systems are drawn into direct conflict) deploy specific sorts of executive-function processes (e.g., cognitive control, which biases information processing toward task-relevant over -irrelevant cues). Moreover, these state-based findings indicate a causal interplay that group comparisons cannot uncover: Increased conflict-processing demands during bilingual comprehension (comprehending a code-switch vs. not) *causes* increased cognitive control during Flanker performance. Similarly, engaging cognitive control from a prior Stroop trial increased bilinguals’ (and monolinguals’) regulation of interpretations during syntactic processing. Thus, comparing bilinguals to themselves under different conditions shows how linguistic and nonlinguistic systems interact and are temporally connected and interdependent from moment to moment. While most research on the relationship between cognitive function and bilingual language concentrates on how cognitive traits are fixed within a group and differ between them, this work reveals how ordinary variations in linguistic context or cognitive engagement *within individuals* also impact processing and performance.

### Future Directions for a States Approach to Bilingualism

We have illustrated some of the questions that States approaches can address and reviewed two state-based study designs: (1) blocking language, task, or pragmatic context to observe the effect on cognitive control (or vice versa), and (2) using trial-to-trial, cross-task conflict adaptation to observe immediate effects of cognitive-control engagement on language processing (or vice versa). As the relationship among bilingualism, executive functions, and their neural correlates remains underspecified, we see these two design options—applied behaviorally and/or with neuroimaging techniques—as viable options for future work that teases apart the role of executive functions in various bilingual language processing contexts. However, these are certainly not the only options for States approach designs.

For example, the extent to which language context affects attention remains underspecified and could be explored using a States approach. Mind-wandering studies indicate that attention is frequently off-task during language comprehension tasks (Boudewyn & Carter, 2018a; Smallwood et al., 2008a; Uzzaman & Joordens, 2011). Future work could manipulate bilingual language context to determine if different language states modulate attention in a mind-wandering paradigm, measuring attentional state through behavioral self-reports or through alpha oscillatory power (cf. Boudewyn & Carter, 2018a). Similarly, Kaan and colleagues demonstrated that the LPC (Moreno et al., 2002)—an ERP component typically found when bilinguals parse a code-switch and reflective of reanalysis or unexpectedness—is reduced when a bilingual participant parses code-switches in the presence of another bilingual interlocutor as compared to a monolingual interlocutor. This suggests that bilingual processing demands are modulated by the pragmatics of a linguistic context (a state) and that cognitive (or attention) control operations may fluctuate on the basis of the interlocutors’ expected shared knowledge. Another possibility would be to determine if prior language context (e.g., code-switched vs. unilingual content) affects the ERP signatures associated with detecting acoustically deviant stimuli in an “oddball” paradigm. For example, a speech disfluency preceding an attention-grabbing, deviant stimulus can reduce the typical mismatch negativity and P300 effects, perhaps because the disfluency had already captured the participants’ attention (Collard et al., 2008). Code-switching may perform a similar attention-capturing function or may lead bilingual listeners to better anticipate less expected or more difficult linguistic information (Tomić & Valdés Kroff, 2021). Such effects may be due to modulations in bilingual comprehenders’ attention based on linguistic context (e.g., tracking a speaker’s state of mind as a window into *why* they might have switched languages mid-utterance).

Moving beyond bilingualism’s effect on various constituents of executive function, it is also reasonable to expect that bilingual experiences may drive recruitment of other types of cognitive and neural machinery. For instance, the integrity of declarative memory systems, supported by the hippocampus, have been shown to influence aspects of online language processing including reference resolution (Kurczek et al., 2013) and the use of common ground to constrain interpretations (Rubin et al., 2011). Given that communication is facilitated when interlocutors exploit shared knowledge (i.e., common ground), it would be well-motivated to investigate whether different bilingual interactional contexts modulate the representation and access of such information to guide comprehension (e.g., common ground among speakers from the same vs. different sociological and linguistic communities). Tracking information from the discourse, such as common- versus privileged-ground information or a pronoun’s antecedent, places pressure on the hippocampal declarative memory system (Duff & Brown-Schmidt, 2012). By extension, the *type* of discourse situation (e.g., code-switching vs. not; communicating with a group member vs. not) may also modulate how much pressure is placed and the extent to which the hippocampus contributes to a linguistic process like common-ground assessment. As before, however, relationships between bilingual experience and cognitive performance should be investigated based on well-developed linking assumptions (e.g., the pragmatic effects sketched above in Kaan et al., 2020).

Alongside these possible approaches, we echo others in calling for clear reporting of the context in which bilingual testing occurs, given the effects of interactional context and particular language experiences (de Bruin, 2019; Luk & Bialystok, 2013; Whitford & Luk, 2019). Recall that bilinguals shift their linguistic and cognitive states in accordance with the demands of a particular situation. In recognizing this immense flexibility, we should adequately describe the interactional context of the larger bilingual community at the site of testing (e.g., Beatty-Martínez et al., 2020) as well as the immediate context of the experiment itself. This includes considering the community’s attitudes toward the languages or toward linguistic behaviors such as code-switching (Dewaele & Wei, 2014; Treffers-Daller et al., 2020), the location of experimental testing along with the associated linguistic norms of that place, and the experimenters with whom the participants interact. Knowing that experimentally manipulated language context can affect a bilingual’s cognitive state (Adler et al., 2020; Jiao et al., 2019, 2020; Wu & Thierry, 2013), we must assume that the language context in the lab (e.g., considering building location, experimenter’s language of choice, experimenter’s register) can affect a bilingual’s cognitive state as well. Indeed, one benefit of a States approach is that these sociolinguistic effects of community or lab context on processing can be investigated. However, when these social implications are not the manipulation of interest, we join others in underlining the importance of reporting in detail the experimental context, because bilinguals (and monolinguals) are surely adjusting to it.

## CLOSING REMARKS

Conventional models of bilingual language processing that describe a role for executive functions overwhelmingly provide correlational data on the individual- or group-level scales, which has demonstrated how aggregate measures of performance compare across bilingual and monolingual populations. Here, we advocate for an emerging and highly compatible approach, which examines the immediate or near-immediate impact of different language contexts on executive function performance and, correspondingly, of different states of cognitive engagement on language processing.

By design, such an approach diverges from the typical one in that experiments within this framework consider individuals against themselves under different linguistic or cognitive states. Rather than provide insights about year-to-year, cumulative differences between groups, this proposal suggests a moment-to-moment timescale that can offer data about the architecture of the language processing system and what roles various cognitive mechanisms play in real time. Necessarily, this requires a shift in the types of narrow research questions that psycholinguists ask (see Box 2). For instance: What are the unique language-processing demands that bilinguals confront during real-time production and comprehension that generate the need for components of executive function to mitigate those demands? Are there idiosyncratic pragmatic inferences a bilingual must draw during communication with other bilinguals versus monolinguals? And, if so, what specific cognitive regulatory mechanisms and associated brain regions does this engage? Do manipulations of attention control (vs. cognitive control) adjust a bilingual’s ability to isolate a speaker’s voice from other sources of noise in a crowded restaurant, avoiding processing difficulty? There is a current gap in knowledge in these areas, but closing it is crucial for advancement toward a complete model of language processing in the bilingual (and monolingual) mind. By manipulating language contexts, one can observe the consequences of those subtle manipulations on particular aspects of cognitive performance; similarly, by manipulating the engagement status of particular cognitive systems (e.g., upregulation of attention control vs. cognitive control vs. some other executive function), with specific linking hypotheses about what language tasks these systems interact with, one can observe the consequences of these subtle manipulations on linguistic performance.

**Box 2.**  Comparing typical states and traits approaches

**Table:** 

	Typical Traits approach	Typical States approach
Example questions approach can address	Do monolinguals and bilinguals recruit executive functions differently?	What moment-to-moment bilingual language-processing demands engage executive functions?
	If so, do bilinguals have an advantage over monolinguals on certain tasks (or vice versa)?	How does a bilingual’s cognitive state impact their language processing (or vice versa)?
Standard methods	Group-comparisons between participants; individual differences studies.	Within-participant comparisons under different conditions (at the block level or at the trial level).
Use of executive functions	Examines individual differences in one-time “snapshot” performance on tasks.	Looks for fluctuations in performance under different conditions of tasks.
Categorizing bilingualism	Dichotomization of “monolingual” and “bilingual” groups to carry out critical between-groups comparisons.	Newer continuous measures of language experience (e.g., entropy) can be incorporated since comparisons are within-groups.
Some advantages	Detects differences that emerge based on cumulative life experiences.	Allows for cause-and-effect claims. Considers an individual’s state of engagement at the time of their performance on a task and the processing demands that task incurs.
Some limitations	Tasks may be poorly suited for detecting individual differences. Splits a continuum of bilingualism into a dichotomy.	Still summarizes an individual’s performance (within each condition). Continues to use artificial, lab-based conditions, which may tap into a specific language mode.

We believe the within-person approach that we have summarized in this article, which has been emerging in psycholinguistic studies of language processing in recent years, is well-suited to address these and a range of other relevant questions and allows causal (rather than correlational) inferences to be drawn about the real-time cognitive and/or linguistic demands that shape performance in the moment. In sum, we suggest that the prevalent Traits approach to bilingual language processing can be usefully complemented with a burgeoning States approach, assessing how different aspects of the bilingual experience rely on executive functions. This should allow us to move beyond discussions of bilingual-monolingual differences toward a better understanding of the processing dynamics of bilingual language processing.

## ACKNOWLEDGMENTS

We would like to thank Gigi Luk and an anonymous reviewer for their helpful feedback.

## FUNDING INFORMATION

Jared M. Novick, National Science Foundation (https://dx.doi.org/10.13039/100000001), Award ID: NSF BCS2020932. Jorge R. Valdés Kroff, National Science Foundation (https://dx.doi.org/10.13039/100000001), Award ID: NSF BCS-2017251. Lauren K. Salig, National Science Foundation (https://dx.doi.org/10.13039/100000001), Award ID: 1449815. Jorge R. Valdés Kroff, National Science Foundation (https://dx.doi.org/10.13039/100000001), Award ID: BCS-1941514.

## AUTHOR CONTRIBUTIONS

**Lauren K. Salig**: Conceptualization: Equal; Writing – original draft: Lead; Writing – review & editing: Equal. **Jorge R. Valdés Kroff**: Conceptualization: Equal; Writing – original draft: Equal; Writing – review & editing: Equal. **L. Robert Slevc**: Conceptualization: Equal; Writing – original draft: Equal; Writing – review & editing: Equal. **Jared M. Novick**: Conceptualization: Equal; Writing – original draft: Equal; Writing – review & editing: Equal.

## TECHNICAL TERMS

Traits: Attributes of a person that guide behavior and remain relatively stable across time and situations (Steyer et al., 2015).
States: Attributes of a person-in-a-situation that fluctuate and involve situation-driven variability (Steyer et al., 2015).
Executive function: The family of cognitive mechanisms that manage information processing in the brain and support the flexible guidance of goal-directed behavior.
Cognitive control: An important sub-component of executive function that specifically detects and resolves conflict during information processing.
Conflict: Situations where task demands require adjudicating among incompatible ways of interpreting a stimulus during information processing (e.g., when the task requires suppressing a dominant/prepotent way of characterizing a stimulus).
Attention control: Another sub-component of executive function that regulates collection of information from the environment.
Conflict adaptation: A behavioral phenomenon where high-conflict trials in tasks like Stroop or Flanker, relative to low-conflict trials, enhance performance on subsequent high-conflict trials.
